# Development of an RT-LAMP−CRISPR/Cas12a assay for rapid and specific detection of *Bandavirus dabieense*

**DOI:** 10.71150/jm.2506013

**Published:** 2025-11-30

**Authors:** Bo Seung Song, Yun Hee Baek, Eun-Ha Kim, Hyeok-Il Kwon, Ah-Hyeon Kim, Si-Hyun Lee, Yu-Bin Son, Soo-Hyeon Kim, Min-Suk Song, Young Ki Choi, Su-Jin Park

**Affiliations:** 1 Division of Applied Life Science, Gyeongsang National University, Jinju 52828, Republic of Korea; 2 Department of Microbiology, College of Medicine and Medical Research Institute, Chungbuk National University, Cheongju 28644, Republic of Korea; 3 Center for Study of Emerging and Re-emerging Viruses, Korea Virus Research Institute, Institute for Basic Science (IBS), Daejeon 34126, Republic of Korea; 4 ChoongAng Vaccine Laboratories, Daejeon 34055, Republic of Korea; 5 Division of Life Science, College of Natural Sciences, Research Institute of Molecular Alchemy (RIMA), Gyeongsang National University, Jinju 52828, Republic of Korea

**Keywords:** RT-LAMP associated CRISPR Cas12a, *Bandavirus dabieense*, diagnosis, SFTS

## Abstract

*Bandavirus dabieense*, a single-stranded RNA virus, is the causative agent of severe fever with thrombocytopenia syndrome (SFTS), a disease associated with high fatality rates. Early and accurate diagnosis is essential for improving clinical outcomes, particularly given the limited therapeutic options and high mortality rates associated with SFTS. However, while highly sensitive, conventional diagnostic methods such as PCR and qRT-PCR require specialized laboratory facilities and trained personnel, making them impractical for rapid detection in resource-limited settings. To address these challenges, we developed a rapid and highly sensitive assay for *Bandavirus dabieense* detection by integrating reverse transcription loop-mediated isothermal amplification (RT-LAMP) with CRISPR/Cas12a technology. LAMP primers and guide RNA sequences were designed to target the L gene, ensuring broad detection across viral genotypes. The optimized assay demonstrated a detection limit of 5 RNA copies per reaction, showing more sensitivity than qRT-PCR, and exhibited 100% concordance with qRT-PCR results in clinical samples. Given its speed, accuracy, and field applicability, this LAMP-CRISPR/Cas12a-based assay represents a promising diagnostic tool for early SFTSV detection, particularly in resource-constrained environments where conventional molecular diagnostics are not readily available.

## Introduction

The *Bandavirus dabieense*, also known as the severe fever with thrombocytopenia syndrome (SFTS) virus or *Dabie bandavirus*, is primarily transmitted through tick bites, particularly from the *Haemaphysalis longicornis* tick, native to China, Korea, and Japan (Jang et al., 2024; Luo et al., 2015; Takahashi et al., 2014). Due to climate change and animal migration, ticks have expanded their range to continents beyond their native regions, including South America, New Zealand, and the Pacific, increasing the global risk of tick-borne diseases like SFTS (Miao et al., 2020). Additionally, the virus can be transmitted not only through tick bites but also through contact with infected body fluids, with a potential risk of airborne transmission, which further contributes to the increased possibility of virus infection (Akagi et al., 2020; Hu et al., 2022; Kim et al., 2015).

*Bandavirus dabieense* has segmented and single-stranded RNA genomes consisting of negative-sense L and M RNA and an ambisense S RNA (Yu et al., 2011). Like other RNA viruses, *Bandavirus dabieense* exhibits high genetic variability, leading to its divergence into six major genotypes (A, B, C, D, E, and F) and further sub-lineages within genotype B (B-1 to B-4) (Fu et al., 2016; Park et al., 2024; Wen et al., 2024). These genotypic variations pose a significant challenge for existing diagnostic methods, necessitating the development of new methods to identify a wide range of viral genotypes.

Conventional nucleic acid-based diagnostics, such as polymerase chain reaction (PCR) and quantitative real-time PCR (qRT-PCR), offer high sensitivity and specificity; however, they require well-equipped laboratory settings and trained personnel, limiting their applicability in resource-limited areas. To overcome these challenges, isothermal amplification methods, including recombinase polymerase amplification (RPA) and loop-mediated isothermal amplification (LAMP), have been developed as alternatives for rapid on-site detection (Craw and Balachandran, 2012; Deng and Gao, 2015; Yan et al., 2014). However, these methods are prone to false-positive results (Gao et al., 2019; Senarath et al., 2014).

To enhance the specificity and reliability, CRISPR/Cas-based diagnostics have been developed, integrating clustered regularly interspaced short palindromic repeats (CRISPR) and CRISPR-associated (Cas) enzymes as highly specific nucleic acid detection systems (Kaminski et al., 2021; Kim et al., 2021). Originally identified as a bacterial adaptive immune mechanism, the CRISPR/Cas system functions by incorporating pathogen-derived nucleic acid fragments into CRISPR loci. Upon subsequent infections, these loci are transcribed into CRISPR RNAs (crRNAs), which guide Cas enzymes in recognizing and cleaving target sequences. In diagnostic applications, Cas12 and Cas13 enzymes exhibit sequence-specific cleavage followed by collateral activity on single-stranded DNA, facilitating highly specific detection (Aquino-Jarquin, 2019; Dronina et al., 2022; Wu et al., 2023; Xu et al., 2024).

Building on these advancements, we developed an LAMP-based detection platform integrating the CRISPR/Cas12a system, achieving highly accurate and sensitive *Bandavirus dabieense* detection. This novel assay provides an accessible tool for early disease intervention and management in resource-limited healthcare settings.

## Materials and Methods

### Viruses, titration, and RNA extraction

All experimental procedures involving *Bandavirus dabieense* (strain: CB1/2014), Severe acute respiratory syndrome coronavirus 2 (SARS‑CoV‑2), Middle East respiratory syndrome-related coronavirus (MERS-CoV), and Hantann virus were conducted in a bio-safety level (BSL) 3 facility at the Institute for Basic Science (permission number KDCA-23-3-06) under the guidelines of the Institutional Biosafety Committee (approval numbers IBS-IBC-2024-29 and IBS-IBC-2024-40). Viruses were propagated in Vero E6 cells, harvested, centrifuged, and stored at -80°C. Virus titration was performed by seeding Vero E6 cells in 96-well plates 24 h prior to infection with tenfold serially diluted viruses. After 72 or 120 h of incubation, the 50% tissue culture infective dose (TCID_50_) was calculated using Reed and Muench's method (Reed and Muench, 1938). RNA was extracted from the propagated viruses using the RNeasy Mini Kit (Qiagen, Germany) following the manufacturer's instructions. The extracted RNA was inoculated into VeroE6 cells to ensure the absence of live viruses. After confirmation, the extracted RNA was transferred to a BSL-2 laboratory for further assays.

Similarly, the influenza virus [strain: A/California/04/2009(H1N1)] and Seoul virus (strain: 80-39) were propagated in Mardin-Darby canine kidney or VeroE6 cells, harvested, centrifuged, and stored at -80℃. Virus titration was also performed as previously described procedure with 48 or 120 h incubation. RNA extraction was performed in a BSL-2 laboratory.

### RT-LAMP primer design and reaction

A total of 1,605 different *Bandavirus dabieense* L gene sequences were downloaded from NCBI GenBank and analyzed using CLC Main Workbench 23 (version 23.0.1) to identify the conserved sequence regions containing the PAM sequence and protospacer sequence within the L gene. Subsequently, the RT-LAMP primer set was designed from the regions using the LAMP primer design tool and further modified for specificity (Table 1). The RT-LAMP reaction was conducted using RT-LAMP 2X Master Mix (Elpis-biotech, Korea) and confirmed by electrophoresing the products on a 2% agarose gel.

### In vitro gRNA synthesis

The guide RNA (gRNA) sequences were designed following the Huang and Cook method (Huang and Cook, 2022). Briefly, a PCR reaction was conducted using the designed gRNA primers (Table 1). A DNA template containing T7 promoter, LbCas12a mature direct repeat, and target nucleotide sequences was amplified by PCR. Subsequently, in vitro transcription was performed on the PCR samples using the EZ MEGA T7 Transcription Kit (Enzynomics, Korea). After transcription, the synthesized RNA was purified with the Monarch RNA Cleanup Kit (NEB, USA) and stored at -80°C until use.

### Cas12a trans-cleavage (reporter-cleavage) assay

The Cas12a trans-cleavage assay was performed using Lba Cas12a (NEB, USA). The reaction mixture, which included buffer, gRNA, and Lba Cas12a, was pre-incubated at 37°C during the RT-LAMP reaction to facilitate the assembly of gRNA and Lba Cas12a. Following this, single-stranded DNA fluorescence-quencher reporter (reporter) and RT-LAMP products were added after the RT-LAMP reaction (Table 1). Fluorescence detection was performed using the CFX96 (Bio-Rad, USA) every other minute at 37°C for 30 min. After the 30-min reaction, the results were also read with light emission using UV light.

### Quantitative RT-PCR

For comparison with RT-LAMP−CRISPR/Cas12a assay, qRT-PCR was performed using the TOPreal SYBR Green RT-qPCR Kit (Enzynomics, Korea) with L gene qRT-PCR primers (Table 1) or the PowerChek^TM^ SFTSV Real-time PCR Kit (KogeneBiotech, Korea), following the manufacturer's instructions. For the TOPreal SYBR Green RT-qPCR Kit, reverse transcription was performed at 50°C for 30 min, followed by initial denaturation at 95°C for 10 min and 35 PCR cycles, consisting of denaturation at 95°C for 10 s and annealing/extension at 60°C for 30 s. For the PowerChek^TM^ SFTSV Real-time PCR Kit, reverse transcription was at 50°C for 15 min, followed by initial denaturation at 95°C for 5 min and 45 PCR cycles. Fluorescence was measured with the M segment detected using JOE and the S segment detected using FAM.

### Clinical samples

RNA was extracted from 14 SFTS-suspected serum samples using the RNeasy Plus Universal Mini Kit (Qiagen, Germany) according to the manufacturer’s protocol. Briefly, 500 µl of Trizol was added to 100 µl of serum and incubated for 5 min. Subsequently, 100 µl of chloroform was added, and the mixture was vortexed for 20 s before being incubated for an additional 3 min at room temperature. The mixture was then centrifuged for 15 min at 4°C, the supernatant was combined with 300 µl of 70% ethanol, and then RNA was purified and stored at -70°C.

### Statistical analysis

Asterisks indicate statistically significant differences between groups as determined by ordinary one-way ANOVA and subsequent Tukey’s multiple comparisons test (** indicates 0.05 < *p* < 0.01, *** indicates 0.01 < *p* < 0.001, and **** indicates *p* < 0.0001). Statistical analyses were performed using GraphPad Prism version 10.00 for Windows (GraphPad Software, USA).

## Results

### Optimization of RT-LAMP

**Fig. 1.**
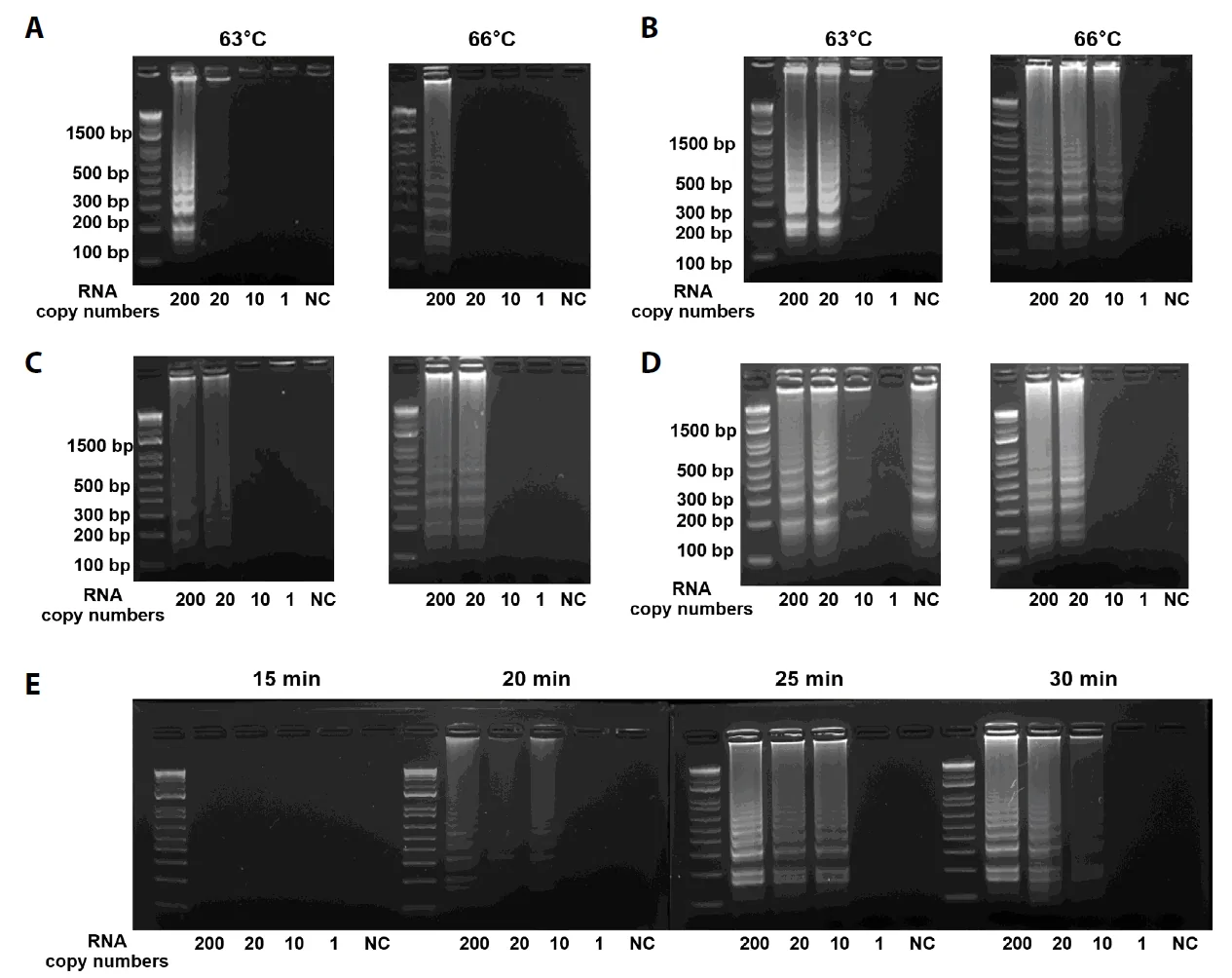
Determination of RT-LAMP reaction conditions. The RT-LAMP reaction was performed under different primer conditions at 63 and 66°C using either 200, 20, 10, or 1 copies of RNA from *Bandavirus dabieense* or distilled water (DW) as a negative control (NC). The specific primer concentrations for each condition were as follows: (A) F3: 0.08 µM, B3: 0.1 µM, FIP and BIP: 0.86 µM, LF: 0.14 µM, and LB: 0.17 µM; (B) F3: 0.18 µM, B3: 0.2 µM, FIP and BIP: 0.86 µM, LF: 0.14 µM, and LB: 0.17 µM; (C) F3: 0.18 µM, B3: 0.2 µM, FIP, and BIP: 0.74 µM, LF: 0.14 µM, and LB: 0.17 µM; (D) F3: 0.18 µM, B3: 0.2 µM, FIP and BIP: 0.86 µM, LF: 0.04 µM, and LB: 0.07 µM; (E) RT-LAMP samples were removed from the heating block and cooled on ice at 15, 20, 25, and 30 min after incubation at 66°C to stop the reaction and assess the reaction time. The RT-LAMP reaction product was analyzed on a 2% agarose gel.

The high sensitivity of the RT-LAMP reaction is essential for the effectiveness of the RT-LAMP–CRISPR/Cas12-based assay. To achieve optimal performance, we designed a set of six RT-LAMP primers (F3, B3, BIP, FIP, LF, and LB) targeting a highly conserved region of the *Bandavirus dabieense* L gene based on sequence data obtained from NCBI (Table 1). Various reaction conditions were systematically tested using RNA extracted from the *Bandavirus dabieense* (Fig. 1).

Various primer concentrations ranged from 0.07 to 0.86 µM (Table 2), and temperatures at 63°C and 66°C were tested for their ability to amplify viral RNA templates at 200, 20, 10, and 1 copies with distilled water (DW) serving as a negative control (NC) (Fig. 1A–1D). Most conditions failed to detect RNA at lower concentrations (10 and 1 copies) or resulted in non-specific amplification (Fig. 1A, 1C, and 1D). However, a specific primer combination—0.18 µM (F3), 0.2 µM (B3), 0.86 µM (BIP and FIP), 0.14 µM (LF), and 0.17 µM (LB)—detected RNA at 10 copies, demonstrating more than twice the sensitivity compared to other primer concentrations (Fig. 1B). While the same primer combination at 63°C also showed bands at 10 copies of viral RNA, detection was inconsistent. Therefore, additional experiments were conducted with this primer combination at 66°C.

Next, the RT-LAMP reaction time was optimized using the established primer concentrations and optimal temperature (66°C) (Fig. 1E). Amplification was detected in samples containing 20 and 10 copies of viral RNA within 20 min. While extending the reaction beyond 20 min increased band intensity, it did not improve the detection limit. Based on these findings, a 20-min reaction time was selected as the optimal condition for RT-LAMP, balancing sensitivity and efficiency for subsequent experiments.

### Optimization of the CRISPR/Cas12a assay

**Fig. 2.**
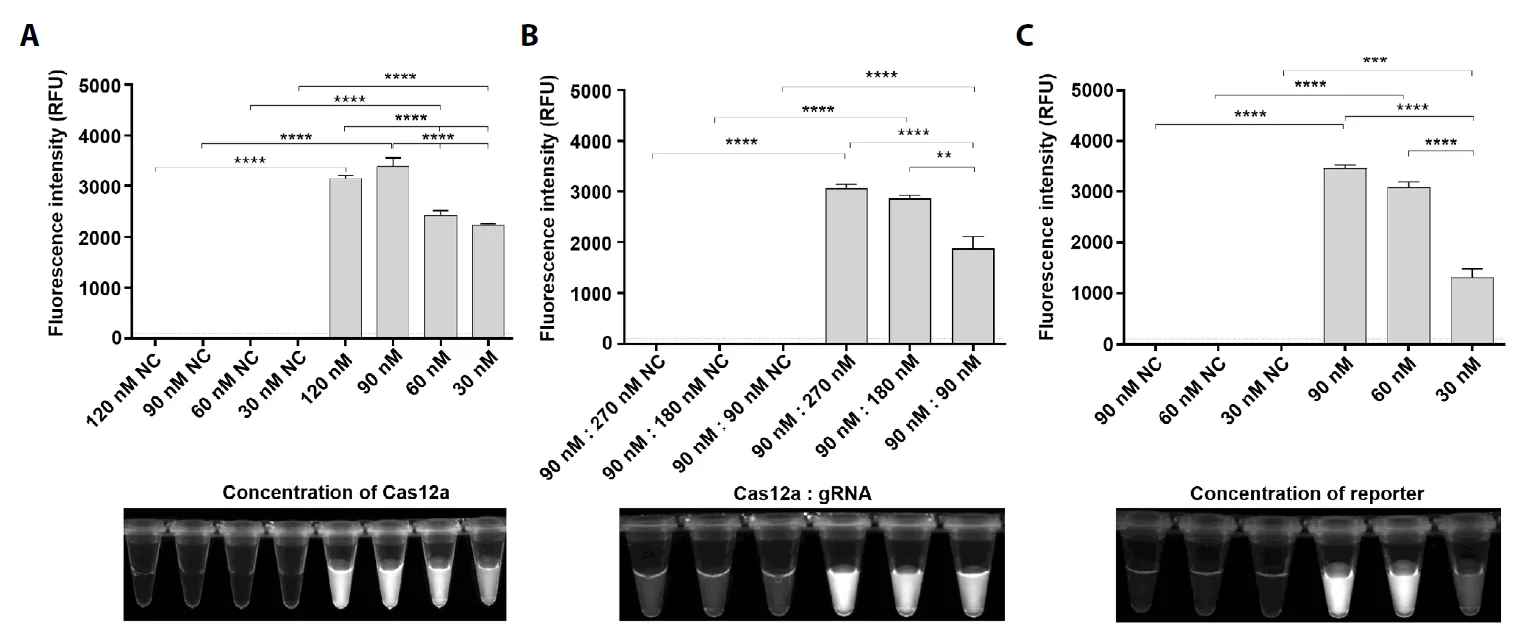
Determination of CRISPR/Cas 12a reaction conditions. The 20 copies of viral RNA or DW were amplified using RT-LAMP, and then these samples were used for optimization of CRISPR/Cas 12a reaction conditions. (A) Optimization of Cas12a concentration, (B) gRNA concentration, and (C) reporter concentration was performed using RT-LAMP products. The results were measured by a real-time PCR machine or visualized by fluorescent intensity under UV light. The fluorescence intensity is presented as Mean ± SEM. Asterisks (*) indicate statistical significance between groups as determined by ordinary one-way ANOVA and subsequent Tukey’s multiple comparisons tests (^**^ indicates 0.05 < *p* < 0.01, ^***^ indicates 0.01 < *p* < 0.001, and ^****^ indicates *p* < 0.0001). NC, negative control.

To maximize the efficiency of the CRISPR/Cas12a-based detection system, we optimized the concentrations of Cas12a, gRNA, and reporter using the 20 copies of viral RNA (Fig. 2). First, we tested a range of Cas12a concentrations (30–120 nM) while maintaining a fixed Cas12a:gRNA ratio of 1:2 and reporter concentration of 60 nM (Fig. 2A). Fluorescence intensity analysis showed that 90 nM Cas12a generated the highest signal, with only a marginal increase at 120 nM. Since both concentrations provided comparable results, 90 nM Cas12a was chosen as the optimal concentration for subsequent experiments. Next, we optimized gRNA concentration by varying its ratio to 90 nM Cas12a (1:1, 1:2, and 1:3). Fluorescence intensity increased with gRNA concentration, stabilizing at 180 nM. As the signal at 180 nM and 270 nM was comparable, we selected 180 nM gRNA as the optimal concentration (Fig. 2B). Finally, we determined the optimal reporter concentration by testing a range of 30–90 nM while keeping 90 nM Cas12a and 180 nM gRNA constant. A strong fluorescence signal was observed at 60 nM, with no significant improvement at higher concentrations (Fig. 2C). Therefore, 60 nM reporter was selected as the optimal condition for subsequent experiments. Further, all samples that did not contain viral RNA demonstrated no fluorescence or light emission under UV light.

### Evaluation of the sensitivity and specificity of RT-LAMP−CRISPR/Cas12a assay

**Fig. 3.**
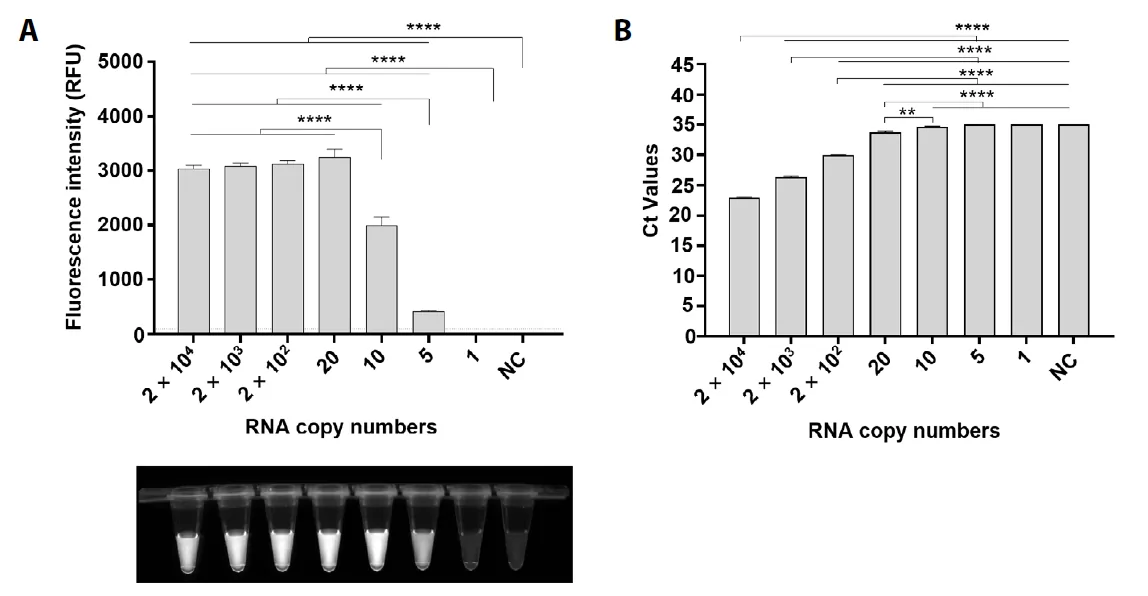
Comparisons of sensitivity in RT-LAMP–CRISPR/Cas12a and qRT-PCR. (A) RT-LAMP-CRISPR/Cas12a assays were conducted using serial dilutions of *Bandavirus dabieense* RNA, with concentrations ranging from 2 × 10^4^ to 1 copy per reaction. The results were measured by fluorescence or visualized based on UV light. (B) The same RT-LAMP-CRISPR/Cas12a assay samples were also used for qRT-PCR targeting the L gene. The fluorescence intensity is presented as Mean ± SEM. Asterisks (*) indicate statistical significance between groups as determined by ordinary one-way ANOVA and subsequent Tukey’s multiple comparisons tests (^**^ indicates 0.05 < *p* < 0.01, ^****^ indicates *p* < 0.0001). NC, negative control.

The assay sensitivity was evaluated by serially diluted *Bandavirus dabieense* RNA, ranging from 2 × 10^4^ to 1 copy per reaction. Fluorescence signals and UV light were detectable at concentrations as low as 5 RNA copies per reaction, establishing a minimum detection limit of 5 copies (Fig. 3A). In comparison, conventional qRT-PCR exhibited a limit of detection of 10 RNA copies per reaction, demonstrating the enhanced sensitivity of the RT-LAMP−CRISPR/Cas12a assay (Fig. 3B). Collectively, these findings confirm that the RT-LAMP−CRISPR/Cas12a assay is a highly specific, sensitive, and reliable diagnostic tool for *Bandavirus dabieense* detection, making it a promising method for rapid viral diagnosis.

**Fig. 4.**
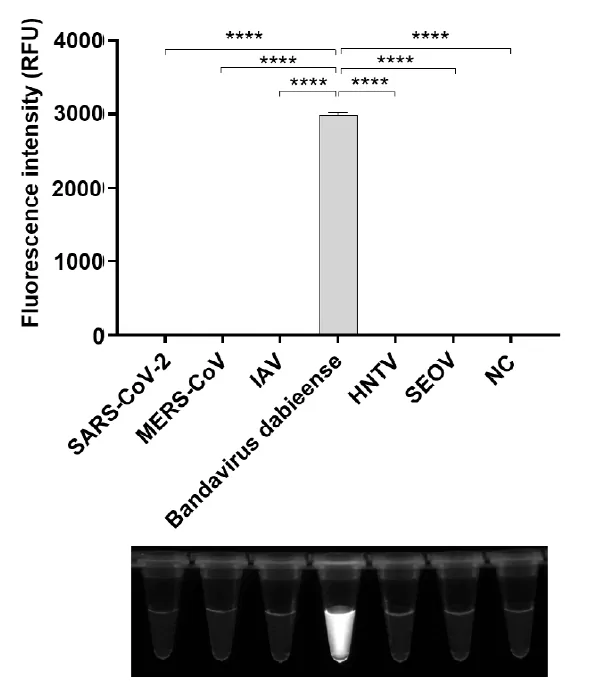
Specificity evaluation of the RT-LAMP−CRISPR/Cas12a detection system. RNA extracted from Severe acute respiratory syndrome coronavirus 2 (SARS‑CoV‑2), Middle East respiratory syndrome-related coronavirus (MERS-CoV), influenza virus (IAV), *Bandavirus dabieense*, Hantann virus (HNTV), and Seoul virus (SEOV) was tested for specificity. For each reaction, RNA was extracted from 100 TCID_50_ of SARS‑CoV‑2, MERS-CoV, IAV, HNTV, and SEOV, while the target RNA from *Bandavirus dabieense* was used at 200 copies per reaction. The results were measured by a real-time PCR machine or visualized by fluorescent intensity under UV light. The fluorescence intensity is presented as Mean ± SEM. Asterisks (*) indicate statistical significance between groups as determined by ordinary one-way ANOVA and subsequent Tukey’s multiple comparisons tests (^****^ indicates *p* < 0.0001). NC, negative control.

We also tested RNA extracted from SARS‑CoV‑2, MERS-CoV, influenza virus, Hantaan virus, and Seoul virus to evaluate the specificity of the developed diagnostic method. Fluorescence signals and light emission were exclusively detected in RNA samples from *Bandavirus dabieense*, with no cross-reactivity observed against other viruses (Fig. 4). These results demonstrated the high specificity of the assay in distinguishing *Bandavirus dabieense* from unrelated viral pathogens.

### Detection of viruses in clinical samples

**Fig. 5.**
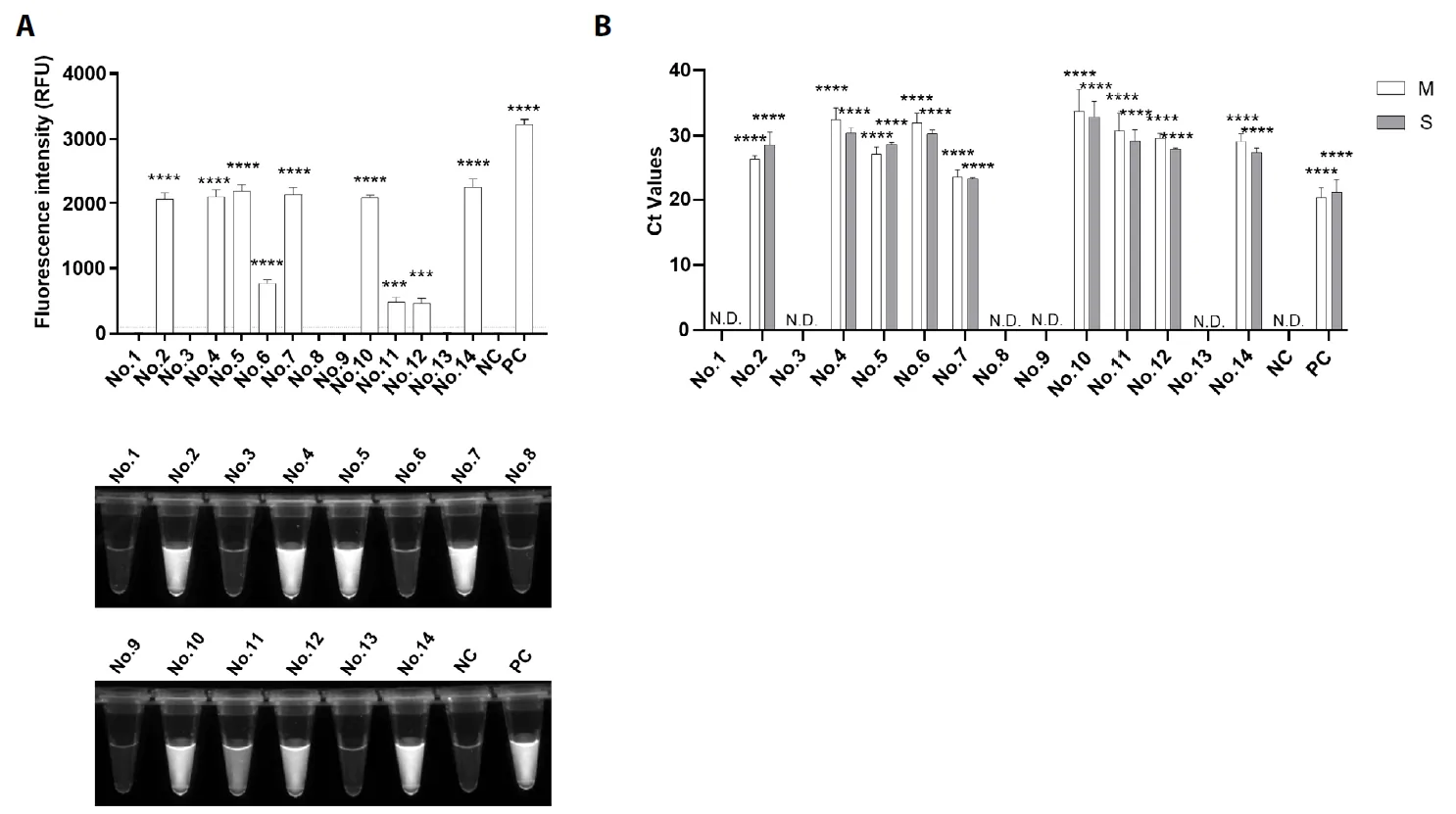
Detection of virus in clinical samples. RNA was extracted from the serum of 14 patients suspected of *Bandavirus dabieense* infection. DW was used as a negative control (NC), while RNA from *Bandavirus dabieense* served as a positive control (PC) sample. (A) RT-LAMP–CRISPR/Cas12a assay was performed with extracted RNA or DW. (B) The identical RNA samples were used for qRT-PCR with M and S gene detection. The results were presented as Mean ± SEM. Asterisks (*) indicate statistical significance between groups as determined by ordinary one-way ANOVA and subsequent Tukey’s multiple comparisons tests (^***^ indicates 0.01 < *p* < 0.001, and ^****^ indicates *p* < 0.0001).

To assess the clinical applicability of the RT-LAMP−CRISPR/Cas12a assay, we tested serum samples from patients suspected of being infected with the *Bandavirus dabieense*. Among the 14 clinical specimens, 9 tested positive, and 5 tested negative using the RT-LAMP−CRISPR/Cas12a method (Fig. 5A). For comparative validation, qRT-PCR targeting the M and S genes was used (KogeneBiotech, Korea). The RT-LAMP−CRISPR/Cas12a assay demonstrated 100% concordance with qRT-PCR results, confirming its diagnostic reliability (Fig. 5B). These results highlight that our developed diagnosis assay provides a reliable and accurate alternative to qRT-PCR for *Bandavirus dabieense* detection in clinical samples, making it a promising diagnostic tool for field applications.

## Discussion

Despite the growing threat posed by the spread of *Bandavirus dabieense*, there are currently no approved vaccines or antiviral drugs specifically for this virus. Clinical studies have suggested that early treatment with favipiravir and ribavirin may improve clinical outcomes in patients with low viral loads upon hospital admission (Jung et al., 2021; Li et al., 2018, 2021). These findings underscore the urgent need for accurate and rapid diagnostic tools to enable timely intervention and improve clinical management of SFTS patients.

Nucleic acid-based detection methods offer high sensitivity and reliability for pathogen identification (Baylis et al., 2019; Niemz et al., 2011). Although qRT-PCR is regarded as the gold standard due to its high sensitivity and reliability, it requires precise temperature-controlled amplification and long reaction times, which are challenging in resource-limited settings (Murphy and Bustin, 2009). In contrast, isothermal amplification techniques enable nucleic acid amplification at a constant temperature, reducing the need for specialized equipment and shortening detection times compared to qRT-PCR (Glökler et al., 2021; Kundu et al., 2024). Additionally, integration of CRISPR/Cas12a into these assays enhances diagnostic accuracy (Kaminski et al., 2021; Kim et al., 2021). Here, we developed an RT-LAMP-based isothermal amplification assay combined with CRISPR/Cas12a detection for rapid and highly specific *Bandavirus dabieense* identification.

The genetic diversity of *Bandavirus dabieense* poses a challenge for consistent diagnostic results (Fu et al., 2016; Park et al., 2024; Wen et al., 2024; Yun et al., 2017). Additionally, the segmented genome allows for reassortment between different viral strains, further increasing genetic diversity (Shi et al., 2017; Wang et al., 2023). To address this, we targeted the L gene, which has a lower variation and slower evolutionary rate compared to the M and S genes, and used mixed-base substitutions in RT-LAMP primers for variant detection (Lee et al., 2023; Yun et al., 2017).

During assay optimization, we identified the optimal primer concentrations for RT-LAMP as follows: F3 (0.18 µM), B3 (0.2 µM), FIP/BIP (0.86 µM), LF (0.14 µM), and LB (0.17 µM) at 66°C. The detection limit remained unchanged when incubation exceeded 20 min, allowing us to set the RT-LAMP reaction time at 20 min. The optimal CRISPR/Cas12a reaction conditions were 90 nM Cas12a and 180 nM gRNA. This RT-LAMP−CRISPR/Cas12a assay achieved rapid detection of *Bandavirus dabieense* in under 50 min, significantly reducing the detection time compared to qRT-PCR.

Our diagnostic method demonstrated no cross-reactivity with SARS-CoV-2, MERS-CoV, and influenza virus, all of which are known to cause high fever in humans, and with the Hantaan virus and Seoul virus, both classified in the order *Bunyavirales*. Additionally, our diagnostic application in clinical samples showed comparable results to those obtained with a commercial qRT-PCR kit, strongly supporting its diagnostic reliability.

While our assay demonstrated high diagnostic accuracy, the limited sample size of 14 clinical specimens suggests the need for further studies with larger and more diverse sample sets to confirm its broader applicability. The genotype distribution data from Korea indicate that multiple *Bandavirus dabieense* genotypes circulate, with genotype B (69–80%) being predominant, while genotypes A, D, E, and F account for 5–8%, 2–4%, 2%, and 4–7%, respectively (Kwon et al., 2024; Yun et al., 2020). Since 9 positive cases were identified, the assay can likely detect multiple genotypes beyond genotype B.

LAMP− and RPA−CRISPR/Cas assays for detecting *Bandavirus dabieense* have demonstrated high specificity and comparable sensitivity in detecting clinical samples suspected of *Bandavirus dabieense* infection while offering reduced reaction times (Huang et al., 2022; Park et al., 2022; Shu et al., 2025). While LAMP is generally more cost-effective than RPA, it requires high temperatures and sometimes lacks specificity (Van Der Hofstadt et al., 2025; Yan et al., 2024). However, when combined with CRISPR/Cas, the specificity issue is effectively increased, though it necessitates using at least two temperature-controlled equipment.

The LAMP and RPA associated with the CRISPR/Cas assay could reduce the non-specificity with shortened reaction time. These characteristics highlight their potential as alternatives to qRT-PCR. These methods are particularly suitable for low-resource or unequipped settings, as they can be conducted using a heat block, water bath, or portable equipment. They also offer the potential for visual readouts using portable UV lights, fluorometers, or integration with lateral flow assays, enhancing their feasibility (Huang et al., 2022; Park et al., 2022). Despite the risk of cross-contamination due to the need to open reaction tubes when using the CRISPR/Cas assay, these methods are cost-effective and reduce operation time compared to qRT-PCR.

In conclusion, our RT-LAMP−CRISPR/Cas12a assay provides a rapid, sensitive, and specific diagnostic tool for *Bandavirus dabieense* detection, offering an alternative to qRT-PCR. Given its high accuracy and field adaptability, this method has significant potential for early SFTS diagnosis in resource-limited settings, facilitating timely disease intervention and management.

## Figures and Tables

**Table 1.** Oligonucleotides used for this study

Primer		Sequences (5` → 3`)	Position
RT-LAMP	F3	GGTGCATGCGAATCTGTCT	2727–2745
	B3	AGCCAGGGCCAAGACT**Y**C	2928–2945
	FIP	TGCACTAGCCGGGCATTAGAATCAGCATGGTGG	2798–2814/2754–2769
	BIP	CATGAGACTGTTGCCAACCCTCTGACTTCAGCCCATGGTT	2861–2881/2906–2924
	LF	GCATCCATAACGTAGAT	2776–2792
	LB	GGCT**Y**AAGAATTC**Y**ATCATAGA	2883–2905
gRNA	F	CCCTAATACGACTCACTATAGG**TAATTTCTACTAAGTGTAGAT**	-
	R	*ACATCTAGCCATGGTCTCAACCC*ATCTACACTTAGTAGAAATTA	-
FQ reporter		FAM-TTATT-BHQ1	-
qRT-PCR	F	ATTCAAGCCTGCCTTAAGTTCAAG	1246–1270
	R	TTTCTTCTGGTTTGCTGCCATT	1321–1342

Mixed base Y = C, T; Underlined, T7 promoter sequence; bold, LbCas12a mature direct repeat; italics, target nucleotide sequences (GGGTTGAGACCATGGCTAGATGT)

**Table 2.** Primer concentration

	F3 (µM)	B3 (µM)	FIP (µM)	BIP (µM)	LF (µM)	LB (µM)
A	0.08	0.1	0.86	0.86	0.14	0.17
B	0.18	0.2	0.86	0.86	0.14	0.17
C	0.18	0.2	0.74	0.74	0.14	0.17
D	0.18	0.2	0.86	0.86	0.04	0.07
